# Report on Variola and Vaccination

**Published:** 1853-02

**Authors:** 


*Report on Variola and Vaccination.* — The committee appointed at the last meeting of the Medical Society of the State of Pennsylvania, to investigate the accuracy of certain views relative to small-pox and vaccination, recently put forth by Drs. Gregory, of London, and Cazenave, of Paris, and referred to in a communication made to the society at its last session, report:

That considering the high authority heretofore attached to the names mentioned, the opinions in question, if erroneous, are calculated to unsettle the views of physicians, and shake the confidence of the public in regard to the protective powers of vaccination, more than any promulgated since its adoption. The committee think these grounds sufficient to justify them in treating the subject with particular attention.

The principal points and questions calling for consideration, are:

1. Whether persons vaccinated, lose, through lapse of time, any of the protective power once afforded against small-pox?

2. Whether the prophylactic powers of vaccination performed during infancy are restricted to the first fifteen years of life and of no avail afterward?

3. Whether the accumulated evidence of the present day is calculated to sustain Dr. Gregory in his belief, that the efficacy of cow-pox as a protection against small-pox has diminished, and a large increase of small-pox resulted from the extension of vaccination?

4. Whether, as asserted by Drs. Gregory and Cazenave, inoculation after the fifteenth year of age, of persons previously vaccinated, produces a specific papular eruptive disease of a non-contagious character, unattended with danger, and giving protection in after life against small-pox?

5. Whether circumstances exist which render it most advantageous to substitute inoculation for vaccination, after the fifteenth year of age, as proposed by Dr. Gregory?

II. The morbid miasm, or agent productive of small-pox, seemed for a long while kept in check by the prophylactic power of vaccination, which, indeed, at one time promised the complete extermination of variola. But it cannot be disputed, that of late years variolous attacks have been common among those hitherto considered as completely protected. A new form of disease has, in fact, become known, designated "*varioloid*," from its resemblance to variola or small-pox, of which it is generally regarded a milder form, as if modified and rendered less formidable, through some remaining prophylactic influence. This, of course, long before Dr. Gregory promulgated his peculiar views, furnished grounds for believing that the protection once relied upon from vaccination, was diminished by lapse of time, or that the potency of the small-pox miasm had increased.

Dr. Gregory's views, when first promulgated in England, were well calculated to rouse the attention of the medical profession, and elicit inquiry. The Epidemiological Society of London, appointed a special committee to investigate the important subjects of vaccination and small-pox, and this committee has recently collected and placed before the public some highly important facts, through its chairman, Mr. Grainger. As the information thus derived is so highly valuable, and directly calculated to meet the points started by Dr. Gregory, the committee think they cannot do better than give a short abstract from Mr. Grainger's statements.

In the evidence brought forward by the committee of the Epidemiological Society, we have the results of the experience of a large number of medical practitioners in different parts of England, and it is interesting to find, that out of 430 replies to questions issued by the society, one, only, expresses any doubt of the protective power of small-pox; and this one doubt simply amounts to this: that having been inoculated during infancy, this gentleman felt himself more secure than if he had been vaccinated!

With regard to opinions founded upon observations prosecuted in hospital practice, the committee would remark, that the results are so often influenced by the existence, here and there, of modifying circumstances, that an appeal to the experience of any single one would certainly afford most incorrect data, on which to found important conclusions, as these should always rest upon multiplied facts, and observations extended through long periods.

In a table presented by Dr. Gregory, and published in his paper given in the *London Medical Times*, for 1849, we find the following statement of the results exhibited in the Small-pox Hospital, over which he presided:

| | Total. | Deaths. | Per centage of Deaths. |
|---|---|---|---|
| Unprotected cases, . . . | 254 | 103 | 40 |
| Vaccinated, with cicatrices, . . | 365 | 38 | 10 |
| " without cicatrices, . | 63 | 25 | 39 |
| Total vaccinated, . . . . | 428 | 63 | 14 |
| Previously inoculated, . . | 3 | 1 | 33 |

Now the rate of mortality here presented, is so much greater than that generally met with, in other institutions or in common practice, as to leave little doubt that the patients had been subjected to some of those malign influences, such as defective ventilation, &c., which have so often operated most injuriously in rendering mild cases severe, and originally severe ones almost inevitably fatal. If we compare the results exhibited in Dr. Gregory's Hospital practice, with those presented in 30 returns received from medical practitioners, by the London Epidemiological Society, taken without selection, we shall find the contrast most striking:

| | Total. | Deaths. | Per centage of Deaths. |
|---|---|---|---|
| Natural small-pox in the unprotected, | 1751 | 361 | 20.85 |
| Small-pox after vaccination, . . | 927 | 32 | 3.44 |

Previous to the introduction of vaccination, the annual mortality from small-pox amounted to 40,000 per annum, in the British Islands alone, being about

1-10th of all the deaths from every source. The average number of deaths per annum in London from small-pox, a century ago, namely, during a decennial period ending in 1750, was 2036; which presents a proportion strongly contrasted with the annual average of a decennial period ending in 1850, which is 498. This shows a mortality four times greater during a period when the population was not a fourth of what it was at the time last named.

Dr. Casper, of Berlin, shows in his statistics that the deaths from small-pox, in Berlin, during the eight years from 1814 to 1822, were 535 out of a general mortality of 51,389, being only 1 death from small-pox in 1000 from all diseases. This exhibits either an almost total absence of epidemic influence, or a very general diffusion of protective means. It is stated in a publication containing the regulations for medical and other officers, issued in Berlin, in October, 1803, that small-pox caused, on an average, 40,000 deaths a year in Prussia, in a population of about 10,000,000, during a period when inoculation was the only protection relied upon. In 1849, when the population had increased to more than 16,000,000, the average mortality from small-pox was 1760; showing that, during the first period, when inoculation was the sole reliance, the proportional mortality from small-pox was 37 times greater than when vaccination became generally diffused. These striking facts are, we think, very far from sustaining Dr. Gregory's opinion that an extension of vaccination has resulted in an increase of small-pox; nor do they offer any encouragement to those who would restore the former practice of inoculation.

The frequent occurrence, of late years, of small-pox after vaccination, with instances of mortality, have been much commented on, and occasioned no small alarm. Hence, the great value of such accurate information as the following, furnished in 356 replies sent by physicians to the Epidemiological Society.

Of these, 182 state expressly that *they have never seen a death* from small-pox after vaccination.

44 state their experience in numbers, and give an aggregate of 70 deaths.

127 give no statements of their experience.

From the same source we gather the results of the experience of 30 physicians on the respective degrees of mortality of natural small-pox, small-pox after small-pox, and small-pox after vaccination.

| | Cases. | Deaths. | Per centage of deaths. |
|---|---|---|---|
| Natural small-pox, . . . | 1731 | 361 | 20.85 |
| Small-pox after small-pox, . | 58 | 22 | 37.92 |
| Small-pox after vaccination, . . | 929 | 32 | 3.44 |

It is remarked, in reference to the 32 deaths reported after vaccination, that in seven cases the evidences of vaccination was not satisfactory, whilst in six other cases the deaths were owing to superadded diseases. Deducting the 13 deaths, the ratio of fatal cases occurring after vaccination would be scarcely two per cent., whereas that of small-pox after small-pox is nearly 38 per cent.

To these statements of results of very extensive experience abroad, we are glad to have it in our power to subjoin evidence equally conclusive as to the protective power of vaccination, obtained among our own practitioners. In the report on varioloid, the protective power of vaccination, &c., presented to the College of Physicians of Philadelphia, in Nov. 1846, replies to interrogatories of the committee were received from 51 practicing physicians of the city and districts, who reported 776 cases of varioloid as having occurred in their practice during the epidemic of that period. Forty of these cases occurred after inoculation or a previous attack of small-pox in the natural way, and the remaining 736 after a reputed vaccination. Of the whole number of 776, cases, but 12 deaths occurred, or less than two per cent., and of these cases several were attended with serious complications. These cases all occurred in private practice, except two, which took place at the Small-pox Hospital, at Bush Hill.

It is worth noticing, among the evidence from abroad upon this subject, that Mr. Marsden, resident physician of the London Small-pox Hospital, has, within the last sixteen years, vaccinated no less than 40,000 persons, not one of whom had returned to the hospital with small-pox. Had there been any considerable number of the vaccinated attacked subsequently with small-pox, there is reason to believe that very many would have found their way to the institution which receive multitudes of patients from the same ranks in which the vaccination took place.

Statements made by Dr. Grainger, prepared from official returns received from all parts of England to the Poor-law Board, show a greater neglect of vaccination than could be well imagined to exist among civilized people. In London, 13 unions, exhibiting 21,598 births, report the number vaccinated at only 4641, or 21 per cent; whilst 31 unions in the country give only 9.2 per cent. vaccinations under the first year of life. In many others, the proportion of infants vaccinated in the first year of life is much less, being occasionally as low as one per cent. Whilst such is the sad case in a country boasting a national vaccine institution, and acts of Parliament for the promotion of vaccination, things seem to be even worse in Ireland. In a very valuable report, made by Mr. Wilson, of Dublin, contained in the report of the census of Ireland for 1841, it is stated that, of the 56,000 deaths from small-pox which occurred in that country in the decennial period from 1831 to 1841, no fewer than 79 per cent., or 45,824, were of children under five years of age. Dr. Gregory gives results for England very nearly the same. He states that of 9762 persons who died of small-pox in that country during the years 1837–38, the deaths under five years were 7340, or about 75 per cent. of the whole. If, as Dr. Gregory asserts, in his valuable lectures on eruptive fevers, the protective power of cow-pox may, for all practical purposes, be considered as complete, at least till the eighth year of life, the frightful infantile mortality here exhibited from small-pox, proves a neglect of vaccination almost equal to that which prevails to such a lamentable extent in Ireland.

In Prussia, Sweden, and some other countries, legislative authority has been brought into play with considerable efficiency in promoting the general extension of vaccination. But still, in despite of every precaution and exertion yet made, it would seem there are everywhere to be found thousands of unprotected persons, among the improvident, ready to become victims to small-pox whenever this may be introduced through epidemic or contagious influences.

In estimating the protective powers of vaccination, the public mind often seizes upon individual and isolated cases of death occurring after vaccination performed in childhood, without forming, at the same time, a just estimate of the vast number of individuals who are thereby enjoying immunity from the ravages of variola. Persons are not given to reflect that such deaths constitute the *exception* to the general law of exemption, and that they happen only among a very few individuals peculiarly susceptible to the variolous poison. It is also highly probable that the limited class upon whom vaccination appears to exert little or no protective power, are rendered no more safe by inoculation or an attack of small-pox, as we find occasional instances of death from a second attack of genuine small-pox, even in persons who have had the disease so severely as to be extensively pitted.

As to the new form of eruptive disease asserted by Drs. Gregory and Cazenave, to be developed by inoculation performed upon those vaccinated previous to the fifteenth year, the committee has been prevented from testing its verity by actual experiments, penal laws existing against inoculating within the city and adjoining districts, embraced within the limits of the Board of Health. A few experiments have, however, been made during the past year by Dr. D. F. Condie, of Philadelphia, on persons situated beyond the jurisdiction referred to, the results of which were by no means calculated to sustain the views of Drs. Gregory and Cazenave. Although such limited experience cannot be regarded as furnishing evidence sufficiently conclusive upon the subject, we think it proper to place the results before the society.

Ten cases were experimented on by inserting variolous matter in the arms of individuals, six of whom had been previously successfully vaccinated by Dr. Condie, and of the successful vaccination of the other four he had the most unquestionable evidence.

In *three* of the cases, between seven and eight years had elapsed since the period of the vaccication.

In *five*, between thirteen and fourteen years.

In *two*, between fifteen and sixteen years.

In *one* case, a local variolous pock appeared upon the arm at the place of inoculation—attended between the eighth and ninth days with a pretty smart fever. The scab separated on the twentieth day, leaving a decided cicatrix. The remaining portion of surface was entirely free from any form of eruption. This individual had undergone successful vaccination seven years and two months previously.

In *four* cases, the local disease was attended with a general eruption of acuminated pocks—with hard base and slight areola—sparsely disseminated over the surface. In different cases from twenty to one hundred pocks appeared. In these cases the pustules on the arm and over the body were attended with a very slight fever about the fifth day—after this period they desiccated very rapidly, forming small, light-brown conical scabs, which commenced falling off on the eighth day, leaving no cicatrix. The periods which had elapsed since vaccination in these cases were, thirteen years in two, fifteen in one, and fifteen years seven months in another.

In *five* cases, a local inflammation, but no pustule, occurred at the part where the matter was inserted, which disappeared within four or six days, leaving no cicatrix. These cases were unattended with fever, or any form of cutaneous eruption. These patients had undergone vaccination seven years and five months, seven years and nine months, thirteen years and six months, and in two between fourteen and fifteen years.

These experiments were performed without the jurisdiction of the Board of Health, of Philadelphia, with the consent of the parties and their friends, and with due precautions to prevent the individuals operated on from becoming foci of contagion.

It certainly appears strange that the poison of small-pox, which, when taken the natural way by persons previously vaccinated, produces the disease in its regular, pustular and contagious form, should, when introduced by inoculation into the systems of persons similarly situated, develop an entirely different form of disease, such as that described by Dr. Gregory as a specific papular eruptive affection of a non-contagious character, unattended with danger, and giving the most perfect protection in after-life against small-pox. Even supposing the result to be as stated by Dr. Gregory, the production of such a mild and benignant train of symptoms as those he describes from the introduction of the small-pox virus, affords one of the strongest evidences of the inestimable protective power exerted by cow-pox.

III. In regard to the fifth and last point of inquiry, your committee have no hesitation in expressing it as their belief, that no circumstances exist to justify the general substitution of inoculation after the fifteenth year of age, as proposed by Dr. Gregory. And they regret that at the present time, whilst strenuous efforts are making through individual exertion, occasionally helped forward by judicious legislation, statements calculated to lessen confidence in the protecting power of vaccination, should have been promulgated. Happily, however, abundant evidence exists to show that although the hopes of complete exemption from small-pox, once fondly indulged, have not been fully realized, vaccination still offers the only dependence for protection against a disease, the fearful ravages of which have tended so much to darken the pages of history previous to the precious discovery made by Jenner.

As the neglect of vaccination, especially among the poor and improvident, may, we think, be regarded as the principal cause operating to promote the extension and mortality of small-pox, the commtttee would urge it upon the State Medical Society to continue their efforts to obtain from the legislature the passage of a law providing for the gratuitous vaccination of the poor, and calculated to secure, as far as practicable, the fullest extension of vaccination in every portion of the commonwealth.

G. EMERSON,
SAMUEL JACKSON,
JOSEPH WARRINGTON,
ISAAC PARRISH,
JOHN D. GRISCOM.

*Trans. Med. Society of State of Penn.*, 1852.

